# Assessment of Growth Factors, Cytokines, and Cellular Markers in Saliva of Patients with Trigeminal Neuralgia

**DOI:** 10.3390/molecules26102964

**Published:** 2021-05-17

**Authors:** Shankargouda Patil, Luca Testarelli

**Affiliations:** 1 Department of Maxillofacial Surgery and Diagnostic Sciences, Division of Oral Pathology, College of Dentistry, Jazan University, Jazan 45142, Saudi Arabia; 2 Department of Oral and Maxillo Facial Sciences, Sapienza University of Rome, 00185 Rome, Italy; luca.testarelli@uniroma1.it

**Keywords:** cytokines, growth factors, salivary biomarkers, trigeminal neuralgia

## Abstract

We proposed to perform a comparative analysis of growth factors, cytokines, and chemokine receptors on the salivary cells in the saliva obtained from trigeminal neuralgia (TN) and normal subjects. Saliva was collected from TN and healthy subjects. Salivary cells were isolated by centrifugation. The expression of the cell surface marker was analyzed by flow cytometry. A cytometric bead array was done to measure the levels of cytokines and growth factors on the flow cytometer. Saliva from TN subjects showed lower growth factor levels of Angiopoietin-2, bFGF, HGF, SCF, TGF-α, and VEGF and higher cytokine levels of IL-1β, TNF-α, CCL2, IL-17A, IL-6, and CXCL8, as well as higher expression levels of chemokine receptors CCR1 (CD191), CR3 (CD11b), CCR2 (CD192), CXCR5 (CD185), and CCR5 (CD196) in the cells from TN saliva. A certain set of cytokines and growth factors in the saliva, as well as chemokine receptors on salivary cells, could be a useful tool in the diagnostics and prognostics of trigeminal neuralgia. Trigeminal neuralgia is one of the significant pathological conditions in the class of chronic diseases around the world. Many targeted approaches are being tried by various research groups to utilize the information of the inflammatory microenvironment to resolve the pathology of chronic TN.

## 1. Introduction

Trigeminal neuralgia (TN) is described as a rare neuropathic condition typified by electrifying facial pain on one side. The pain is usually restricted to the trigeminal area of the face [1]. These painful episodes may last for a few days to several months, for which the trigger can be chewing, touch, or even talking but the exact cause is still under study [2]. Earlier TN was known by the name tic douloureux [3]. TN is classified as classical trigeminal neuralgia (CTN) and secondary trigeminal neuralgia (STN) which can be caused by physical compressions of a trigeminal nerve root due to a tumor or any abnormal artery. Demyelination of the neurons is another reason that triggers TN [4]. Both the categories might have different trigger points but the pain caused is sudden and extreme [5]. The pain symptom associated with TN usually affects older adults and incidences increase with age. Certain studies have also tried to link TN with genetics [6]. The occurrence of cases in clusters within a family indicates a potential genetic etiology for classic trigeminal neuralgia. In this regard, few anomalies including inherited anatomical changes influencing the skull base leading to vascular structure induced trigeminal nerve compression have been suggested. In addition, there are reports of familial AHT where trigeminal nerve compression is induced by tortuous vessels. Calcium channel gene mutation can also cause hyperexcitability. The presence of TN in older age disrupts the quality of life because of the pain episodes [7]. Moreover, unnecessary dental procedures were performed to deal with TN when not much was known about this [1,8].

The primary treatment of TN is antiepileptic medication, sodium channel blockers, and, depending on the pain, certain other drugs can be added. The next sort of treatment is surgery which involves compression of the nerve [2,9,10]. The diagnosis is mainly done through the analysis of symptoms along with the patient history but clinicians suggest that nuclear magnetic resonance (MRI) can help in confirming the diagnosis [4,11]. The trigger points of TN have also been suggested to be used as a diagnostic method [1].

Migrating monocytes towards inflamed or injured tissue is known for creating a long sustainable inflammatory microenvironment in many chronic inflammatory diseases as they have chemokine receptors, including CCR2, CXCR5, CCR5, and CCR1, to migrate along with CCL2 and CCL3 gradients as well as secreting many inflammatory cytokines and chemokines like CCL2, IL-8, IL-6, IL-1β and TNF-α [12]. Periodontal inflammatory microenvironment mainly consists of pro-inflammatory cytokines, including IL-1Beta, TNF-alpha, IL-6, IL-17, and IL-8, which are secreted by cells like monocytes, macrophages, and other cells of the adaptive and innate immune system. Many targeted approaches are being tried up by various research groups to utilize the information of the inflammatory microenvironment to resolve the pathology of chronic TN pain.

Growth factors also play an important role in the development of TN. Lee et al. [13] proved through their study that the vascular endothelial growth factor (VEGF) pathway contributes to the development of TN. Another mechanism suggested by Taylor & Ribeiro-da-Silva [14] revolves around glial cell line-derived growth factor (GNDF) which is elevated in the nerve lesions. Elevation in the insulin-like growth factors (IGF-1) in the nerve bundles followed by any injury or compression has also been shown to be evident [15]. Nerve growth factor (NGF) mediates the signaling associated with TN and can be useful in managing TN pain by acting as a therapeutic target [16].

Following the studies conducted earlier on cytokines and growth factors, this study aims at providing several points which can surely contribute to both the diagnosis and management of TN. We have analyzed the levels of various growth factors in the saliva of healthy and TN-affected subjects. Further, we studied several cytokines like IL-6, IL-10, and IL-1β in both healthy and affected subjects. Clear differences in the levels of growth factors and cytokine will make it evident that they can potentially provide new insights into the treatment and diagnosis. Saliva has been considered as a non-invasive tool for the diagnosis of several conditions in comparison with serum [17]. Alterations in the levels of salivary cytokines of oral cancer have been reported [18,19]. However the salivary levels of cytokines and growth factors in Trigeminal Neuralgia have not been assessed so far. With the available information, the present study was conducted to assess the growth factors, cytokines, and chemokine receptors on the salivary cells in the saliva obtained from trigeminal neuralgia (TN) and normal subjects.

## 2. Results

**Figure 1:**
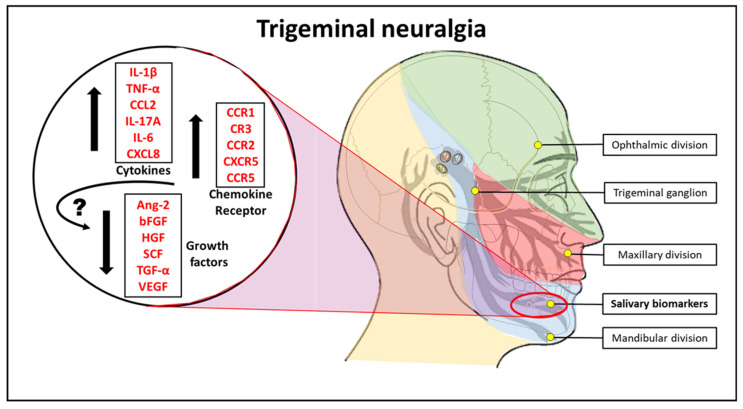
Pictorial abstract of the study. IL-4: Interleukin 4, IL-2: Interleukin 2, CXCL10: C-X-C motif chemokine ligand 10, IL-1β: Interleukin 1 beta, TNF-α: Tumour necrosis factor-alpha, CCL2: C-C motif chemokine ligand 2, IL-17A: Interleukin 17A, IL-6: Interleukin 6, CXCL8: Interleukin 8, VEGF: Vascular endothelial growth factor, TGF-α: Transforming growth factor-alpha, SCF: Stem cell factor, HGF: Hepatocyte growth factor, bFGF: Basic fibroblast growth factor, Ang-2: Angiopoietin-2, CCR1: C-C chemokine receptor type 1, CR3: Complement receptor 3, CCR2: C-C chemokine receptor type 2, CXCR5: C-X-C chemokine receptor type 5, CCR5: C-C chemokine receptor type 5.

Figure 1 is the graphical abstract summarizing the study findings.

### 2.1. Saliva from TN Subjects Show Lower Growth Factor Levels of Angiopoietin-2, bFGF, HGF, SCF, TGF-α, and VEGF than Saliva from Normal Subjects

**Figure 2:**
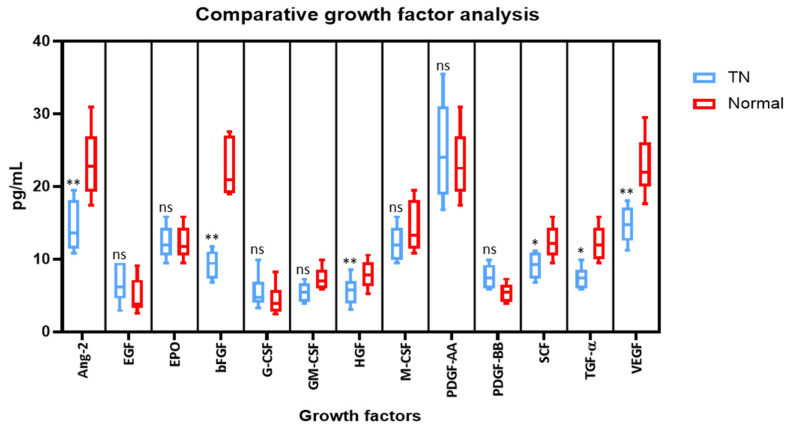
Comparative analysis of growth factors in the saliva of subjects with and without trigeminal neuralgia by cytometric bead array on a flow cytometer. * *p* < 0.05, ** *p* < 0.01. VEGF: Vascular endothelial growth factor, TGF-α: Transforming growth factor-alpha, SCF: Stem cell factor, PDGF-AA: Platelet-derived growth factor AA, PDGF-BB: Platelet-derived growth factor-BB, HGF: Hepatocyte growth factor, bFGF: Basic fibroblast growth factor, EPO: Erythropoietin, EGF: Epidermal growth factor, Ang-2: Angiopoietin-2, G-CSF: Granulocyte colony-stimulating factor, GM-CSF: Granulocyte-macrophage colony-stimulating factor, M-CSF: Macrophage colony-stimulating factor, ns means not significant.

Cytometric bead array was performed to compare the protein levels of growth factors in the saliva (Ang-2, EGF, EPO, bFGF, G-CSF, GM-CSF, HGF, M-CSF, PDGF-AA, PDGF-BB, SCF, TGF-α, and VEGF). Compared to the saliva from normal subjects, the levels of Angiopoietin-2, bFGF, HGF, SCF, TGF-α, and VEGF were significantly lower in the saliva of TN subjects. Whereas, there were no significant changes in the levels of EGF, EPO, G-CSF, GM-CSF, M-CSF, PDGF-AA, and PDGF-BB in both cases (Figure 2).

### 2.2. Saliva from TN Subjects Show Higher Cytokine Levels of IL-1β, TNF-α, CCL2, IL-17A, IL-6, and CXCL8 than Saliva from Normal Subjects

**Figure 3:**
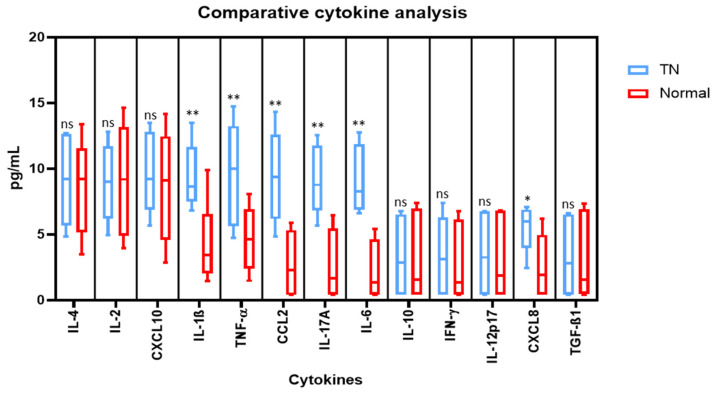
Comparative analysis of cytokines in the saliva of subjects with and without trigeminal neuralgia by cytometric bead array on a flow cytometer. * *p* < 0.05, ** *p* < 0.01. IL-4: Interleukin 4, IL-2: Interleukin 2, CXCL10: C-X-C motif chemokine ligand 10, IL-1β: Interleukin 1 beta, TNF-α: Tumour necrosis factor alpha, CCL2: C-C motif chemokine ligand 2, IL-17A: Interleukin 17A, IL-6: Interleukin 6, IL-10: Interleukin 10, IFN-γ: Interferon gamma, IL-12p70: Interleukin 12, CXCL8: Interleukin 8, TGF-β1: Transforming growth factor beta 1, ns means not significant.

Comparative cytokine analysis was performed by cytometric bead array to assess the protein levels of cytokines in the saliva (IL-4, IL-2, CXCL10, IL-1β, TNF-α, CCL2, IL-17A, IL-6, IL-10, IFN-γ, IL-12p70, CXCL8, TGF-β1). In comparison to the normal saliva, the levels of IL-1β, TNF-α, CCL2, IL-17A, IL-6, and CXCL8 were found to be significantly higher in the saliva from TN subjects. However, no noteworthy changes were observed in the levels of IL-4, IL-2, CXCL10, IL-10, IFN-γ, IL-12p70, and TGF-β1 in both types of saliva samples (Figure 3).

### 2.3. Cell Isolated from the Saliva of TN Subjects Exhibit Higher Marker Expression for Chemokine Receptors CD191, CD11b, CD192, CD185, and CD196 than the cells from the Saliva of Normal Subjects

**Figure 4:**
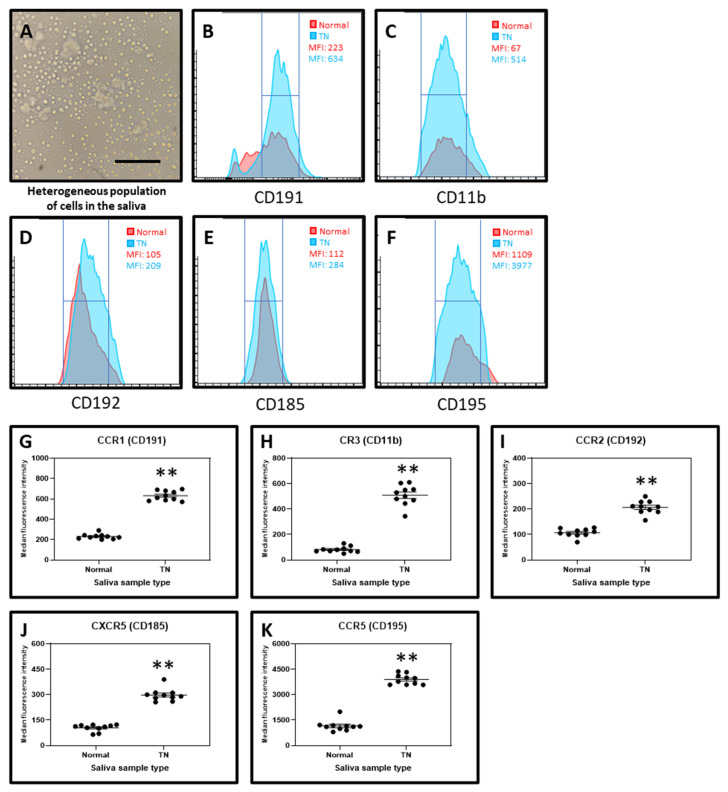
Chemokine receptor analysis by flow cytometry. (**A**) Cells isolated from saliva. Scale bar = 40 μm. (**B**) Median fluorescence intensity (MFI) of CD191 in the salivary cells from normal and TN subjects. (**C**) MFI of CD11b in the salivary cells from normal and TN subjects. (**D**) MFI of CD192 in the salivary cells from normal and TN subjects. (**E**) MFI of CD185 in the salivary cells from normal and TN subjects. (**F**) MFI of CD195 in the salivary cells from normal and TN subjects. (**G**–**K**) Comparative markers analysis of salivary cells from normal and TN subjects of chemokine receptors CD191, CD11b, CD192, CD185, and CD195. ** *p* < 0.01. CCR1 (CD191): C-C chemokine receptor type 1, CR3 (CD11b): Complement receptor 3, CCR2 (CD192): C-C chemokine receptor type 2, CXCR5 (CD185): C-X-C chemokine receptor type 5, CCR5 (CD196): C-C chemokine receptor type 5.

Cellular content was obtained by centrifugation of saliva samples and subjected directly to antibody staining and acquisition on a flow cytometer. The chemokine receptors CCR1 (CD191), CR3 (CD11b), CCR2 (CD192), CXCR5 (CD185), and CCR5 (CD196) were found to be highly expressed in the cells from the saliva of TN subjects and their expressions were significantly higher than in the cells from the saliva of normal subjects (Figure 4).

## 3. Discussion

There is a need to delineate pain biomarkers to improve the quality-of-life of patients with the recent advances in molecular medicine that have increased in the recent past given the advent of personalized/precision medicine techniques and the need to improve the overall quality of life in patients. The 2018 workshop of international experts in pain research by the U.S. National Institutes of Health and National Institute of Neurological Disorders and Stroke recommended the optimal practices in pain biomarker discovery and validation. The objective of the meet was to provide a clear definition for pathophysiologic pain subsets and to evaluate novel drug target engagement. It also aided in prognosing the novel drugs analgesic efficacy. Despite the numerous measures by the conference proceeding, the state of pain biomarker detection and quantification remains vague. Up until now, pain management including facial pain is largely based on self-report by patients and clinical diagnosis and decision making. Among the various conditions manifesting orofacial pain, trigeminal neuralgia is a condition characterized by stabbing or electric-shock-like pain in the regions of the face innervated by the trigeminal nerve. The duration of pain is for a few seconds and occurs unilaterally, sometimes accompanied by spasm of the facial muscles. The maxillary and the mandibular division of the trigeminal nerve are often affected in TN. In such cases, the effect is more severe in on right side than on the left side of the face. TN with bilateral presentation is relatively rare, thus such a presentation must lead to a suspicion of an underlying neurologic disease or cranium affecting non-neurologic. Women are more frequently affected than men, with the risk increasing with age.

Considering management of the condition, certain drugs or chemicals that interfere with the metalloproteinases (MMP-9/2) have proven to be successful in alleviating TN if diagnosed early [20,21,22]. A mechanism proposed by Trevisan et al. [23] states that the released by-products of the monocytes and macrophages in TN induces oxidative stress leading to constriction of the infraorbital nerve that causes pain. This mechanism was also reflected in their study. TN diagnosis and management involves a multi-specialty approach with the involvement of medical (neurology, neuroradiology), surgical (neurosurgery, dentistry, maxillofacial surgery), and pain specialists. Thus, a TN classification must entail all the differential diagnoses frequently noted in these specialties. The significance of accurate diagnosis can be reflected by the customized treatment required by a patient with classical TN (pain relieved by medication) and secondary TN patients (pain relieved by invasive treatment). The evidence level is not differentiated by the ICHD, with the latest ICHD edition not recognizing secondary TN as a diagnostic label. Painful TN is instead used to label TN with major neurologic disease. Distinct conditions including acute herpes zoster induced TN are also included in this label. In order to avoid further confusion, the more widely accepted label of secondary must be maintained.

Considering the prognosis, several studies have shown that the disease has a poor prognosis with deterioration over time [24]. Studies related to TN have always been performed in relation to the nerve, its compression, and surgical methods for treatment. However, considering the poor prognosis of the condition, recent research interest has shifted towards assessing the role of cytokines and growth factors in the disease that may influence the treatment and prognosis. With the available data, the present study was conducted to assess and compare the levels of growth factors, cytokines, and chemokine receptors on the salivary cells in the saliva obtained from trigeminal neuralgia (TN) with normal individuals.

In the present study we observed that the levels of Angiopoietin-2, bFGF, HGF, SCF, TGF-α, and VEGF were significantly lower in the saliva of patients with TN in comparison with the control group. Studies have shown that low levels of VEGF play a role in neuronal degeneration which is a characteristic feature of trigeminal neuralgia [25]. Similarly, angiopoietin- 2 also promotes neovascularization along with VEGF. Hence low levels of VEGF in the present study could be correlated with the neuronal degeneration in TN [26]. Considering the decreased levels of bFGF, HGF, SCF, and TGF-α inpatients with TN in comparison with normal individuals, this could be attributed to the fact that these factors possess neuroprotective function and prevent neuronal degeneration [27,28,29,30]. This is the first study to have assessed these levels, and further studies with a larger sample size have to be carried out to establish the exact role of these markers.

In the present study we observed elevated levels of IL-1β, TNF-α, CCL2, IL-17A, IL-6, and CXCL8 in the saliva from TN subjects. The findings are concurrent with the findings of Liu et al. who reported elevated levels of IL-6, IL-8, IL-1β, and TNF-α, and proposed that the compression of nerve roots in TN is responsible for the secretion of cytokines in their blood [31]. Additionally, IL- 6 has been directly shown to be involved in the pain sensations associated with neuropathic pain [16,17]. It has also been shown that increased levels of CXCL2 (chemokine ligand 2, C-X motif) and IL-10 contribute to the pain induced in the cases of TN. A recombinant administration of both CXCL2 and IL-10 can positively reduce pain and hence they can act as a therapeutic point in the management and diagnosis of TN [32]. Dauvergne and colleagues [33] proposed that CCL2 (chemokine ligand 2, C-C motif) comes into an immediate picture after pain episodes, and hence contribute to the facial hypersensitivity which is a characteristic of TN. Further, macrophages and their secretions have been linked up with nerve injuries, therefore, it can be speculated that TN will have a similar case [12]. Similarly, RANTES or CCL5 is included in the list of cytokines directly involved in TN. The signaling pathways that can be traced concerning these ligands and cytokines might also provide new treatment methods [21]. However on the present study there was no significant difference in the levels of IL-4, IL-2, CXCL10, IL-10, IFN-γ, IL-12p70, and TGF-β1. This could be attributed to the low sample size of the study, and further studies with larger sample size have to be conducted to determine the exact role of these markers in trigeminal neuralgia. Thus, these inflammatory markers could be further explored for targeted therapy as well as determination of prognosis.

Considering the levels of chemokine receptors, we found that CCR1 (CD191), CR3 (CD11b), CCR2 (CD192), CXCR5 (CD185), and CCR5 (CD196) were higher in TN patients in comparison with the control group. Several studies have reported the role of these chemokine receptors in neuropathic pain. Hence the increased expression of these chemokine receptors plays a vital role in pain pathogenesis of TN [34,35,36,37,38]. Thus, these chemokine receptors could be explored as therapeutic target for TN. Though we were able to study the secretory protein levels of various cytokines and growth factors, and expression levels of chemokine receptors, further investigations are needed to correlate these complex molecular interactions to understand the root cause of pain to develop better therapeutic strategies. Also, this study needs to be refined with a larger sample size and careful inclusion/exclusion criteria.

## 4. Materials and Methods

### 4.1. Sample Collection and Ethical Permissions

Ethical permissions required for this study were obtained from the Scientific Research (IRB) College of Dentistry, Jazan University (Reference No. CODJU-19751), and the saliva samples were collected from the subjects (*n* = 20, of which TN = 10 (Age: 32–45 years) and normal = 10 (Age: 26–38 years)) along with the patient informed consent. Inclusion criteria for case group were patients above 18 years of age who were diagnosed with trigeminal neuralgia according to the guidelines reported by Cruccu et al. [20]. Patients who had other systemic diseases, under long-term medications for diseases, children, pregnant women, lactating women and patients who were already on treatment for trigeminal neuralgia were excluded from the study. For the control group, inclusion criteria were patients above 18 years of age who were systemically healthy and were not under medications for any conditions were included. Exclusion criteria for the control group were patients who were having systemic diseases, pregnant and lactating women, and children. The saliva samples were collected under the tongue (unstimulated) (~0.5 mL) in a sterile Eppendorf tube and transported directly to the laboratory for immediate experimental analysis.

### 4.2. Isolation of Cells from Saliva and Flow Cytometry Analysis

The saliva samples were diluted with phosphate-buffered saline (PBS) (1:10) and centrifuged at 1000 rcf for 10 min. The pellet obtained was then further subjected to antibody staining and flow cytometry analysis. Briefly, the cells were resuspended in PBS and labeled with anti-CD191-PE (1:100 dilution), anti-CD11b-APC (1:100 dilution), anti-CD192-APC (1:100 dilution), anti-CD185-PE (1:100 dilution), and anti-CD195-FITC (1:100 dilution) (Miltenyi Biotec, Bergisch Gladbach, Germany). After incubation for 30 min at room temperature, the cells were acquired on a flow cytometer (Attune NxT, Thermo Fisher Scientific, Waltham, MA, USA). A total of 5000 cell events were acquired in each sample. The median fluorescence intensities were calculated and compared.

### 4.3. Cytometric Bead Array for the Detection of Cytokines and Growth Factors

A cytometric bead array was performed to determine the levels of the cytokines and growth factors in the saliva. LEGENDplex™ Human Growth Factor Panel (13-plex) (BioLegend, San Diego, CA, USA) (Angiopoietin-2, EGF, EPO, bFGF, G-CSF, GM-CSF, HGF, M-CSF, PDGF-AA, PDGF-BB, SCF, TGF-α, and VEGF) was used for the detection of the growth factors. LEGENDplex™ Human Essential Immune Response Panel (13-plex) (BioLegend, San Diego, CA, USA) (IL-4, IL-2, CXCL10, IL-1β, TNF-α, CCL2, IL-17A, IL-6, IL-10, IFN-γ, IL-12p70, CXCL8, TGF-β1) was used for the detection of the cytokines. The further experimental protocol was performed according to the manufacturer’s guidelines. Briefly, 25 μL of the saliva sample was incubated with the microbeads for 2 h. After the incubation, the detection antibodies were introduced subsequently to the tests and incubated for 30 min. Further, the samples were washed with wash buffer and centrifuged at 650 rcf for 5 min. The supernatant was removed and the pellet was resuspended in 200 μL sheath fluid. The samples were then acquired on a flow cytometer (Attune NxT, Thermo Fisher Scientific, Waltham, MA, USA) to detect fluorescence intensities, and analysis was performed by manual calculations for individual growth factor or cytokine.

### 4.4. Statistical Analysis

The results were shown as the mean ± standard deviation of the values from all the experimental values. The data were analyzed by using an unpaired *t*-test (two-tailed) on GraphPad Prism 8 software (GraphPad Software, La Jolla, CA, USA) for each cytokine, and *p* < 0.05 was measured as significant (* *p* < 0.05 and ** *p* < 0.01).

## 5. Conclusions

In the present study patients with trigeminal neuralgia showed lower levels of growth factor levels such as Ang-2, bFGF, HGF, SCF, TGF-α, and VEGF and higher cytokine levels of IL-1β, TNF-α, CCL2, IL-17A, IL-6, and CXCL8 in comparison with normal individuals. Also, a higher expression levels of chemokine receptors CCR1 (CD191), CR3 (CD11b), CCR2 (CD192), CXCR5 (CD185), and CCR5 (CD196) were present in in the cells from the saliva of individuals with TN in comparison with normal subjects. With the limitations of the study, it can be concluded that increased levels of chemokines and chemokine receptors play a vital role in pain pathogenesis and a decreased expression of growth factors could be associated with neuronal degeneration in TN. Further investigation is required to not only to identify the assess the biomarkers for diagnosis and prognosis TN but also develop the drug targets for the management of the condition, thereby improving the prognosis of the disease.

## Data Availability

Not applicable.

## Sample Availability

Samples of the compounds are not available from the authors.
